# Association among β2‐adrenergic receptor autoantibodies and proximal left anterior descending artery lesions in patients with initial ST‐segment elevation myocardial infarction

**DOI:** 10.1002/clc.24129

**Published:** 2023-08-17

**Authors:** Wenxi Dang, Ning Cao, Yue Zhang, Weiping Li, Hongwei Li

**Affiliations:** ^1^ Department of Cardiology, Cardiovascular Center, Beijing Friendship Hospital Capital Medical University Beijing China; ^2^ Beijing Key Laboratory of Metabolic Disorder Related Cardiovascular Disease Beijing China; ^3^ Laboratory of Clinical Medicine Capital Medical University Beijing China

**Keywords:** primary percutaneous coronary intervention, proximal left anterior descending, ST‐segment elevation myocardial infarction, β2‐adrenergic receptor autoantibody

## Abstract

**Background:**

β_2_‐adrenergic receptor autoantibody (β_2_‐AA) are widely present in patients with many different types of cardiovascular diseases. Proximal left anterior descending (LAD) artery lesions are associated with adverse prognostic events in patients with ST‐segment elevation myocardial infarction (STEMI).

**Hypothesis:**

β_2_‐AA is associated with the presence of proximal LAD lesions in patients with STEMI.

**Methods:**

A cohort of 153 patients with STEMI who underwent primary percutaneous coronary intervention (PPCI) was enrolled in the study. Baseline characteristics were compared between the proximal LAD group (*n* = 62) and the nonproximal LAD group (*n* = 91). Admission serum of patients was collected to detect the level of β_2_‐AA. Data for echocardiogram within 24 hours after PPCI and at the 6‐month follow‐up were recorded.

**Results:**

The optical density values and positive rates of β_2_‐AA in the proximal LAD group were higher than those in the nonproximal LAD group (*p* < 0.05). β_2_‐AA positively correlated with high sensitivity C‐reactive protein and peak N‐terminal pro‐B type natriuretic peptide levels in the proximal LAD group, but those were not relevant in the nonproximal LAD group. Multivariate logistic regression analysis revealed that high β_2_‐AA levels was independently associated with the presence of proximal LAD lesions in patients with STEMI. Furthermore, a receiver operating characteristic curve was used to show the efficiency of β_2_‐AA levels to detect proximal LAD lesions, and the AUC of the β2‐AA OD value was 0.658 (95% confidence interval 0.568−0.749; *p* = 0.001).

**Conclusions:**

The STEMI patients with high β_2_‐AA levels had a greater possibility having proximal LAD lesions.

## Graphical abstract

The level of β_2_‐AA as a risk factor had value in independently predicting proximal left anterior descending artery (LAD) lesions in patients with ST‐elevation myocardial infarction, patients with high β_2_‐AA were more likely to have the proximal LAD lesions.

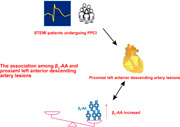

## 1 INTRODUCTION

Acute ST‐segment elevation myocardial infarction (STEMI) results from acute, severe, and persistent ischemia or hypoxia following the blockage of a coronary artery. It may lead to life‐threatening adverse events, such as electrical instability, remodeling or dysfunction.^1^ The left anterior descending artery (LAD) is considered high risk for coronary heart disease because it supplies a considerable area of the myocardium. Several studies have shown that proximal LAD lesions are associated with a higher incidence of adverse outcomes in malignant ventricular arrhythmias and STEMI.^2^ The location of the culprit lesion is one of the important factors in determining the clinical outcome of STEMI. Therefore, it is important to locate proximal LAD lesions in patients with STEMI.

STEMI may cause sympathetic nervous system excitation, and the excited sympathetic nervous system leads to a surge in the release of catecholamines, which act on the cardiac β adrenergic receptor (β‐AR). The β_2_‐adrenergic receptor autoantibody (β_2_‐AA), a G protein‐coupled receptor autoantibody (GPCR‐AA), binds and continuously activates the β_2_‐adrenergic receptor (β_2_‐AR), which causes a variety of pathophysiological reactions. Hence, β_2_‐AA is involved in numerous cardiovascular diseases, such as heart failure, arrhythmia, and orthostatic hypotension. In addition, this strong binding and subsequent stabilization of the adrenergic receptor by its autoantibodies lead to the occurrence and deterioration of heart failure.^3^ However, few studies have focused on the β_2_‐AA level in patients with STEMI and explored the relationship between β_2_‐AA and coronary artery lesions.

Thus, this study compared the differences between proximal LAD lesions and nonproximal LAD lesions. We explored the relationship between β_2_‐AA levels and proximal LAD lesions in STEMI patients.

## 2 METHODS

### 2.1 Study population

Between October 2019 and November 2021, a total of 158 patients consecutively diagnosed with STEMI undergoing PPCI who met the inclusion and exclusion criteria were enrolled in this study from the Beijing Friendship Hospital of Capital Medical University. This study was approved by the Ethics Committee of Beijing Friendship Hospital, Capital Medical University, in accordance with the Declaration of Helsinki. All patients signed informed consent forms. The inclusion criteria were as follows: (1) met the diagnostic criteria for STEMI; and (2) underwent PPCI and accepted drug‐eluting stent (DES). The exclusion criteria were as follows: (1) patients with previous myocardial infarction or coronary revascularization; (2) patients with atrial fibrillation; (3) patients with renal insufficiency (GFR < 30 mL/min/1.73 m^2^); (4) patients with glaucoma; and (5) patients with acute infectious diseases within the past 3 months or rheumatic immune system diseases or malignant tumors. According to the results of coronary angiography, the patients were included in the proximal LAD group and the nonproximal LAD group. Venous blood was collected immediately after admission and stored at −80°C until further analysis. An echocardiogram was performed within 24 hours after PPCI. The results of an echocardiogram taken at 6 months after discharge were reviewed.

### 2.2 ELISA

The OD values of β_2_‐AA was determined by ELISA using synthetic peptides that correspond to the sequence of the second extracellular loop of the human β_2_ receptors (amino acid sequence number 172−192: H‐ W‐ Y‐ R‐ A‐ T‐ H‐ Q‐ E‐ A‐ I‐ N‐ C‐Y‐ A‐ N‐ E‐ T‐ C‐ D‐ F‐ F‐ T‐ N‐ Q). ELISA was performed as stated by previous study.^4^ Briefly, microtiter plates were coated with peptide at 10 µg/mL in coating buffer. The serum samples (dissolved in 1% bovine serum albumin) were added to the microtiter plates. Then, peroxidase‐conjugated anti‐human IgG was diluted 1:500 in the same buffer and added to the plates. Secondary antibodies dilution ratio (ZSGB‐BIO, ZB‐2304): 1:500. Thereafter, a peroxidase substrate ammonium salt was added to the wells and allowed to react for 30 minute. The optical density (OD) values were measured using a microplate reader and were evaluated at 405 nm. The titer was determined by the ratio of P/N (positive/negative controls) as follows: P/N = (sample OD—blank OD)/(healthy control OD—blank OD). Autoantibody positivity or negativity was judged by P/N > 2.1 or P/N < 1.5.

### 2.3 Statistical analysis

All statistical analyses were performed using SPSS 25.0. (IBM). Continuous variables are presented as the mean ± standard deviation (SD) or median (25th quartile, 75th quartile). Student's *t* test or the Mann–Whitney *U* test was used for comparisons between two groups. Categorical data are expressed as percentages (%), and the chi‐square test or Fisher's exact test was used for comparisons between two groups. Correlations between β_2_‐AA and parameters were assessed using Pearson or Spearman tests. Univariable and multivariable logistic analyses were used to determine the effect of the covariates on proximal LAD lesions. Receiver‐operating characteristic (ROC) curve analysis was performed to determine the ability of β_2_‐AA for predicting proximal LAD lesions. A *p* < 0.05 was considered statistically significant.

## 3 RESULTS

### 3.1 Baseline characteristics

**Figure 1:**
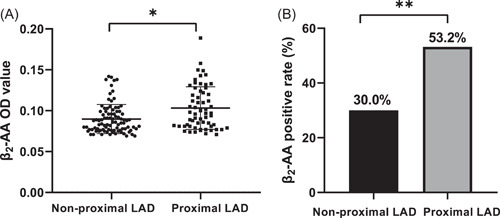
Comparison of the β_2_‐AA OD value and positive rate in LAD and nonproximal LAD groups. Comparison of the β_2_‐AA OD values in the nonproximal LAD and proximal LAD groups. The β_2_‐AA OD value in the proximal LAD group was higher than that in the nonproximal LAD group. (B) Comparison of the β_2_‐AA positive rate in the two groups. The β_2_‐AA‐positive rate in the proximal LAD group was higher than that in the nonproximal LAD group. *p < 0.05, **p < 0.01. LAD, left anterior descending.

Five (3.3%) patients were excluded from the study because of death and loss to follow‐up. A total of 62 patients were included in the proximal LAD group, and 91 patients were included in the nonproximal LAD group according to the intervention site. The baseline characteristics of the two groups are presented in Table 1. Compared with the nonproximal LAD group, patients in the proximal LAD group showed a higher percentage of stroke and higher fasting blood glucose (FBG) (all *p* < 0.05). Comparing the level of β_2_‐AA in the two groups, we found that the OD values and positive rates of β_2_‐AA in the proximal LAD group were higher than those in the nonproximal LAD group (Figure 1). By stratifying patients with proximal LAD and nonproximal LAD lesions into subgroups based on β_2_‐AA OD values, patients with proximal LAD lesions were more frequently distributed in the higher quartiles of β_2_‐AA OD values (Supporting information: Figure S1).

**Table 1:** Baseline and clinical characteristics of the study population.

	Proximal LAD (*n* = 62)	Nonproximal LAD (*n* = 91)	*p* Value
Male, *n* (%)	54 (87.1%)	71 (78.0%)	0.154
Age, year	59.68 ± 10.72	57.57 ± 11.68	0.260
BMI, kg/m^2^	25.94 ± 3.37	25.78 ± 3.72	0.790
Previous history			
Hypertension, *n* (%)	38 (61.3%)	60 (65.9%)	0.557
Diabetes mellitus, *n* (%)	21 (33.9%)	28 (30.8%)	0.686
Hyperlipemia, *n* (%)	29 (46.8%)	40 (44.0%)	0.731
Stroke, *n* (%)	11 (17.7%)	6 (6.6%)	0.031
Current smoker, *n* (%)	47 (75.8%)	65 (71.4%)	0.548
LM/three vessel lesions, *n* (%)	42 (67.7%)	50 (54.9%)	0.112
Laboratory values			
Peak NT‐proBNP, pg/mL	1467.0 (878.00−3026.25)	1439.00 (653.00−2440.00)	0.922
Peak CK‐MB, ng/mL	142.50 (94.00−363.50)	182.40 (93.70−294.00)	0.669
Peak TNI, ng/mL	50.00 (26.80−50.00)	50.00 (20.58−50.00)	0.823
HsCRP, mg/L	6.65 (2.95−19.23)	4.50 (2.20−14.40)	0.121
WBC, 109/L	9.18 ± 2.58	9.19 ± 2.56	0.987
FBG, mmol/L	6.75 (25.68−9.13)	6.10 (5.30−7.50)	0.032
HbA1c, %	5.90 (5.50−7.28)	5.90 (5.40−6.80)	0.584
eGFR, mL/min/1.73 m^2^	99.30 ± 18.26	98.45 ± 20.34	0.792
Creatinine, µmol/L	70.15 (58.90−82.73)	70.10 (57.70−81.60)	0.922
Total Cholesterol, mmol/L	4.85 (4.18−5.53)	4.80 (4.20−5.50)	0.972
Triglyceride, mmol/L	1.50 (1.20−2.30)	1.70 (1.02−1.29)	0.107
LDL‐C, mmol/L	2.90 (2.50−3.40)	2.90 (2.50−3.40)	0.972
ApoA1, g/L	1.15 (1.03−1.28)	1.17 (1.02−1.29)	0.769
ApoB, g/L	0.87 (0.73−1.07)	0.86 (0.73−1.00)	0.972
ApoE, g/L	3.66 (3.21−4.74)	3.92 (3.19−4.68)	0.669
LP(a), mg/L	116.5 (40.75−264.50)	95.00 (49.00−200.00)	0.373
Treatment preadmission			
Antiplatelet angents, *n* (%)	3 (4.8%)	8 (8.8%)	0.353
ACEI/ARBs, *n* (%)	16 (25.8%)	24 (26.4%)	0.938
Beta‐blockers, *n* (%)	7 (11.3%)	3 (3.3%)	0.103
Statins, *n* (%)	38 (61.7%)	3 (4.12%)	0.194

*Note*: Values are presented as mean ± SD, median (25th−75th quartiles) or *n* (%). The p values were obtained using Student's *t*‐test, Mann–Whitney *U*, *χ* ^2^, or Fisher's exact tests.

Abbreviations: ACEI/ARB, angiotensin converting enzyme inhibitor/angiotensin II receptor blocker; ApoA1, Apolipoprotein A1; ApoB, Apolipoprotein B; ApoE, Apolipoprotein E; BMI, body mass index, CK‐MB, creatine kinase‐MB; eGFR, estimated glomerular filtration rate; FBG, fasting blood glucose; HbA1c, glycated hemoglobin; HsCRP, high sensitivity C‐reactive protein; LDL‐C, low‐density lipoprotein cholesterol; LP(a), Lipoprotein(a); NT‐proBNP, N‐terminal pro‐B type natriuretic peptide; TNI, troponin I; WBC, white blood cell.

### 3.2 Correlations of β_2_‐AA in proximal LAD and nonproximal LAD lesions

**Figure 2:**
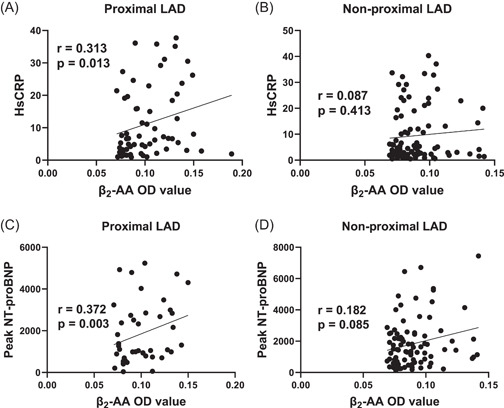
Correlation analysis of β_2_‐AA in LAD and nonproximal LAD groups. (A, B) β_2_‐AA was related to hsCRP in the proximal LAD group. (C, D) β_2_‐AA is related to peak NT‐proBNP in the proximal LAD group. HsCRP, high sensitivity C‐reactive protein; LAD, left anterior descending; NT‐proBNP, N‐terminal pro‐B type natriuretic peptide.

We compared the relationship between the level of β_2_‐AA and baseline data in the proximal LAD and nonproximal LAD groups, and β_2_‐AA was related to N‐terminal pro‐B type natriuretic peptide (NT‐proBNP) and high sensitivity C‐reactive protein (hsCRP) in all patients (Supporting information: Table S1). Moreover, β_2_‐AA was positively correlated with hsCRP and peak NT‐proBNP in the proximal LAD group (*r* = 0.313, *p* = 0.013; *r* = 0.372, *p* = 0.003), but it was not relevant in the nonproximal LAD group (Figure 2).

### 3.3 The predictors of proximal LAD lesions

The results of the logistic regression analyses are shown in Table 2. In the univariate logistic regression analysis, the β_2_‐AA positivity rate and stroke were associated with proximal LAD lesions, and after multivariate logistic regression analysis, the β_2_‐AA positivity rate remained the only independent predictor of proximal LAD lesions after correction. for other baseline determinants (odds ratio [OR] [95% confidence interval (CI)]: 2.88 [1.32−6.30], *p* = 0.008). Meanwhile, the ROC curve was used to show the efficiency of β_2_‐AA levels to measure proximal LAD lesions, and the AUC of the β_2_‐AA OD value was 0.658 (95% CI 0.568−0.749; *p* = 0.001) (Supporting information: Figure S2). The comparison results of the echocardiogram for patients in the two groups at the 6‐month follow‐up are shown in Table 3. At baseline, the diameter of the left atrium (LA) in the nonproximal LAD group was higher than that in the proximal LAD group (*p* = 0.012). Compared with baseline and 6‐month follow‐up, cardiac structure parameters such as LA, left ventricular end‐diastolic diameter (LVEDD), left ventricular end‐diastolic volume (LVEDV), and LVEDV index (LVEDVi) were increased at the 6‐month follow‐up (all *p* < 0.05). Cardiac function parameters, such as LVEF, also were increased at the 6‐month follow‐up (*p* < 0.001).

**Table 2:** Logistic regression analyses of proximal LAD lesion.

	Univariable analysis		Multivariable analysis	
	OR (95% CI)	*p* Value	OR (95% CI)	*p* Value
β2‐AA positivity rate	2.80 (1.31−6.02)	0.008	2.88 (1.32−6.30)	0.008
Age, year	0.53 (0.22−1.29)	0.259		
Male	1.02 (0.99−1.06)	0.158		
BMI, kg/m^2^	1.01 (0.93−1.11)	0.788		
Hypertension	0.82 (0.42−1.60)	0.557		
Diabetes mellitus	1.15 (0.58−2.30)	0.687		
Hyperlipemia	1.12 (0.59−2.14)	0.731		
stroke	3.06 (1.07−8.76)	0.038	3.61 (0.99−13.02)	0.055
current smoker	1.25 (0.60−2.62)	0.549		
Treatment preadmission				
Antiplatelet angents	0.53 (0.13−2.08)	1.360		
ACEI/ARBs	0.97 (0.47−2.03)	0.938		
Beta‐blockers	3.73 (0.93−15.05)	0.064		
Statins	2.33 (0.63−8.63)	0.205		
FBG, mmol/L	1.08 (0.96−1.22)	0.183		
HbA1c, %	1.02 (0.84−1.25)	0.812		
eGFR, mL/min/1.73 m^2^	1.01 (0.99−1.02)	0.790		
Total cholesterol, mmol/L	1.02 (0.76−1.38)	0.893		
Triglyceride, mmol/L	0.87 (0.64−1.18)	0.363		
LDL‐C, mmol/L	1.02 (0.64−1.61)	0.934		
ApoA1, g/L	0.89 (0.18−4.49)	0.892		
ApoB, g/L	1.87 (0.46−7.56)	0.379		
ApoE, g/L	1.00 (0.99−1.01)	0.390		
LP(a), mg/L	1.09 (0.89−1.34)	0.955		
HsCRP, mg/L	1.02 (0.99−1.05)	0.280		

*Note*: Odds ratios (ORs) and p values are shown where the variable was included in the multivariable analysis.

Abbreviations: ACEI/ARB, angiotensin converting enzyme inhibitor/angiotensin II receptor blocker; ApoA1, Apolipoprotein A1; ApoB, Apolipoprotein B; ApoE, Apolipoprotein E; BMI, body mass index, CK‐MB, creatine kinase‐MB; FBG, fasting blood glucose; HbA1c, glycated hemoglobin; HsCRP, high sensitivity C‐reactive protein; LDL‐C, low‐density lipoprotein cholesterol; LP(a), Lipoprotein(a).

**Table 3:** Comparison of 6‐month follow‐up results of echocardiography.

	Proximal LAD (*n* = 62)	Nonproximal LAD (*n* = 91)	*p* Value
LA, cm			
Baseline	3.80 (3.50−4.00)	3.90 (3.60−4.20)	0.012
6 M	3.95 (3.66−4.19)	3.95 (3.72−4.20)	0.873
*p* Value baseline versus 6 M	0.006	0.125	
LVEDD, cm			
Baseline	4.90 (4.70−5.22)	5.00 (4.70−5.30)	0.252
6 M	5.16 (4.80−5.47)	5.13 (4.87−5.41)	0.715
*p* Value baseline versus 6 M	0.007	0.072	
LVESD, cm			
Baseline	3.50 (3.20−3.80)	3.50 (3.20−3.80)	0.965
6 M	3.48 (3.24−3.79)	3.48 (3.23−3.89)	0.801
*p* Value baseline versus 6 M	0.869	0.686	
LVEDV, mL			
Baseline	112.81 (101.60−135.34)	118.24 (102.36−135.34)	0.416
6 M	127.21 (107.39−145.44)	125.50 (111.21−141.92)	0.707
*p* Value baseline versus 6 M	<0.001	0.034	
LVESV, mL			
Baseline	50.52 (40.20−61.95)	47.44 (39.42−58.13)	0.922
6 M	50.17 (42.06−61.37)	50.17 (41.89−65.51)	0.772
*p* Value baseline versus 6 M	0.521	0.282	
LVEDVi, mL/m^2^			
Baseline	128.17 (106.90−154.82)	133.81 (117.29−153.16)	0.277
6 M	141.64 (124.17−170.84)	142.91 (120.77−166.93)	0.922
*p* value baseline versus 6 M	0.011	0.081	
LVESVi, mL/m^2^			
Baseline	53.25 (44.98−69.01)	55.53 (46.40−68.88)	0.922
6 M	58.82 (46.56−69.84)	56.78 (47.54−73.80)	0.575
*p* Value baseline versus 6 M	0.506	0.332	
LVEF, %			
Baseline	50.49 ± 8.27	51.64 ± 6.74	0.347
6 M	56.46 ± 7.14	55.36 ± 7.90	0.382
*p* Value baseline versus 6 M	<0.001	<0.002	

*Note*: Values are mean ± SD, median (upper quartile, lower quartile). The *p* Values were obtained from Student's *t* test, Mann‐Whitney *U* test.

Abbreviations: LA, left atrium diameter; LVEF, left ventricular ejection fraction; LVEDD, left ventricular end‐diastolic diameter; LVESD, left ventricular end‐systolic diameter; LVEDV, left ventricular end‐diastolic volume; LVESV, left ventricular end‐systolic volume; LVEDVi, left ventricular end‐diastolic volume index; LVESVi, left ventricular end‐systolic volume index.

## 4 DISCUSSION

In this study, we found that high β_2_‐AA levels were independently associated with the presence of proximal LAD lesions in patients with STEMI. In addition, patients with STEMI who had proximal LAD lesions had higher NT‐proBNP and higher hsCRP levels, and these patients showed worse cardiac function and inflammatory responses.

The LAD artery has an important position because it supplies 45% to 55% of the left ventricle mass. The PROTECT (Patient Related Outcomes with Endeavor vs. Cypher Stenting Trial) study showed that proximal LAD lesions were associated with a higher incidence of myocardial infarction during long‐term follow‐up.^5^ The study showed that the proximal LAD group had a higher incidence of major adverse cardiovascular events and all‐cause mortality than the nonproximal LAD group.^6^, ^7^ The TWENTE I‐III study also found that the rate of the device‐oriented composite clinical endpoint (cardiac death, target vessel myocardial infarction, or target lesion revascularization) was higher in proximal LAD lesions.^8^ The SYNTAX (Synergy between PCI with Taxus and Cardiac Surgery) score was also significantly higher in the proximal LAD group than in the nonproximal LAD group.^7^ These results indicated that ischemia caused by proximal LAD lesions put more myocardium at risk and lead to a worse prognosis. However, the SORT‐OUT II study found opposite results. The 10‐year mortality rates were similar for patients with proximal LAD and nonproximal LAD stents. The incidence of combined clinical endpoints such as cardiac death, myocardial infarction, or target revascularization was low in the proximal LAD group.^9^ While these results were controversial, we can explain these differences. By comparing the risk characteristics of previous studies, we found that patients with many comorbidities or an extensive history of PCI may have received coronary artery bypass grafting instead of PCI at the time of study inclusion. As a result, such patients may be less likely to participate in randomized PCI trials. The NOBLE study also demonstrated that the differences in outcomes may not be driven by the lesions of interest (e.g., the left main or proximal LAD) but by other new pathologic changes.^10^ In addition, parts of the differences from these trials were the different interventions. Which is more suitable for PCI or coronary artery bypass grafting (CABG) in patients with proximal LAD lesions has always been the focus of controversy. With the development of science and technology, the increasing maturity of PCI and the widespread use of DES, the mortality rate of STEMI patients after PCI has decreased, but the complications of postoperative life crisis are still troubling us. Unlike the patients included in some of the studies above, our research subjects were all STEMI patients in this study, and they were at higher bleeding and thrombotic risks. The more complex pathological and surgical features of proximal LAD patients, who had more multivessel therapy, bifurcation lesions, and severe calcified lesions and who had longer stents, suggest the presence of more extensive coronary artery disease. Therefore, patients with lesions at the proximal end of the LAD represent a subgroup of patients at higher risk.

The interaction between the sympathetic nervous system and the parasympathetic system also plays important roles. The β‐adrenergic receptor (AR) accounts for 90% of the total AR in the heart.^11^ β_2_‐AR is one of the components related to the pathogenesis of various cardiovascular diseases. However, few previous studies have explored the level of β_2_‐AA in STEMI patients. In the process of STEMI, the excited sympathetic nervous system will cause the secretion of a large amount of catecholamines, which will act on the β_2_‐AR of the heart, resulting in the continuous activation of β_2_‐AR, resulting in a series of adverse phenomena. Therefore, inhibiting the continuous activation of β_2_‐AR is very important to reduce the cardiac injury and improve the prognosis of patients with acute myocardial infarction.^12^, ^13^ Previous study showed that β_2_‐AA had ability to antagonize the effects imposed by β_1_‐AA both in vitro and in vivo. β_2_‐AA seems to be a protective factor to some extent in patients with HF.^14^ Actually, all of this is due to the fact that the activation of β_2_‐AR has two sides. β_2_‐AR can conjugate the excitatory G protein (Gs) or inhibitory G protein (Gi). Under the stimulation of catecholamine at normal physiological concentrations, β_2_‐AR activates the downstream protein kinase A (PKA) pathway by coupling the Gs protein to participate in cardiac contraction. When catecholamines are continuously stimulated at high concentrations, activated PKA and G protein‐coupled receptor kinase phosphorylate β_2_‐AR, promoting the conversion and activation of the coupled Gs protein to the Gi protein. Activation of the β_2_‐AR/Gi pathway can inhibit the PKA pathway, thereby inhibiting the overcontraction and apoptosis of cardiomyocytes.^15^, ^16^ The β_1_‐Gs‐mediated effect is partially attenuated by the β_2_‐Gi effect.^4^ Simultaneously, β_2_‐AA also interferes with receptor circulation and desensitization, resulting in receptor passivation or sensitization to endogenous catecholamines. These changes lead to the deterioration of HF. The study showed that enhanced activation of β_2_‐AA leads to sustained atrial tachycardia in rabbits.^17^ The activation of β_2_‐AA may contribute to systemic vasodilation, which may lead to or exacerbate orthostatic hypotension.^18^ Experiments in vitro showed that when the oxygen supply of cardiomyocytes was optimal, β_2_‐AA was inactive. After lactic acid was added, the cells showed full activity against β_2_‐AA.^4^ The above results caused us to think that β_2_‐AR may be abnormally exposed under abnormal conditions such as ischemia and hypoxia (or, e.g., myocardial infarction), leading to the formation of β_2_‐AA. Moreover, inflammation is important in the course of myocardial infarction. Autoimmune processes are increasingly recognized as the origin or amplification of various diseases. Autoantibodies induce a regular immune response that leads to the destruction of the affected tissue.^4^ We found that β_2_‐AA was related to NT‐proBNP and hsCRP. NT‐proBNP is an independent predictor of HF readmission after ACS.^19^ HsCRP is involved in triggering immune processes such as vascular remodeling and plaque deposition, and it is associated with an increased risk of cardiovascular diseases.^20^ Ozluk et al showed that patients with proximal LAD lesions had a significant increase in the neutrophil to lymphocyte ratio (NLR) at admission.^1^ This demonstrated the application of inflammatory markers in the localization of coronary arteries. Our study also explored the relationship between GPCR‐AA and coronary lesions. We found that β_2_‐AA can predict proximal LAD artery lesions in patients with STEMI. Compared with the nonproximal LAD lesions, the proximal LAD lesions had higher levels of β_2_‐AA. β_2_‐AA remained the only independent predictor of proximal LAD lesions. This is the first study to correlate the levels of autoantibodies and coronary anatomy, and the localization of culprit lesions is important for the clinical diagnosis and long‐term prognosis of STEMI. In addition, β_2_‐AA can be used as a tool to locate culprit plaques in patients with STEMI.

Cardiac remodeling may occur in 20%−32% of patients with STEMI, despite successful TIMI level 3 recanalization of infarct‐related arteries. It is also associated with the development of heart failure and worse long‐term clinical outcomes.^21^ Although PCI and DES may reduce the restenosis rate and some risk of these lesions, the incidence of heart failure associated with myocardial infarction remains significant.^22^ Our study compared the difference in echocardiogram‐related indicators at baseline and a 6‐month follow‐up, and we found that the differences between the two groups were not large at baseline. Parameters such as LA, LVEDD, LVEDV, and LVEDVi were increased at the 6‐month follow‐up. Considering that the proximal LAD supplies a considerable area of the myocardium, it makes sense that the proximal LAD lesions had changed the cardiac structure. In addition, cardiac function parameters, such as LVEF, were restored. These studies showed that drug treatment significantly improved cardiac function and remodeling, and carvedilol was able to reduce the frequency and titer of β_2_‐AA in patients.^18^, ^23^ In our study, all enrolled subjects received optimal discharge guidance during follow‐up, and they were all given an individualized medication regimen. All of the above results showed that effective treatment can improve cardiac function and prognosis and help us identify new therapeutic targets.

In summary, we found that patients with high β_2_‐AA levels had a greater possibility having proximal LAD lesions. and can influence left ventricular remodeling, which may further affect the long‐term prognosis. The discovery of β_2_‐AA will provide new therapeutic targets and ideas.

### 4.1 Limitations

This study has some limitations. It was a single‐center study that cannot comprehensively cover different types of patients, and the sample size was relatively small. In addition, our study is observational in nature, the surgical techniques were based on operator choice, and the treatment and management of all subjects were based on the attending physicians. All subjects had fewer clinical events because of the good follow‐up management. In the future, we need to include larger sample sizes with more clinical follow‐up data.

## 5 CONCLUSIONS

The level of β_2_‐AA as a risk factor had value in independently predicting proximal LAD lesions in patients with STEMI, and can provide novel therapeutic targets in the future.

## CONFLICT OF INTEREST STATEMENT

The authors declare no conflict of interest.

## Supporting information

Supporting information.

## Data Availability

The data sets used and analyzed in this study are available from the corresponding authors upon reasonable request.
