# Supplementary material for: Association among β2‐adrenergic receptor autoantibodies and proximal left anterior descending artery lesions in patients with initial ST‐segment elevation myocardial infarction
Source: Clin Cardiol. 2023 Aug 17;46(11):1371–9. doi: 10.1002/clc.24129 (PMC10642316; doi:10.1002/clc.24129)
Supplement: Supplementary file 1 — Supporting information. [file CLC-46-1371-s001.docx]

**SUPPLEMENT APPENDIX**

**Table S1. Correlation analysis between β_2_-AA and baseline parameters.**

|  |  | Correlation (r) | *p* value |  |
| --- | --- | --- | --- | --- |
| peak NT-proBNP, pg/ml |  |  |  |  |
|  | Total | 0.277 | <0.002 |  |
|  | Proximal LAD | 0.372 | 0.003 |  |
|  | Non-proximal LAD | 0.182 | 0.085 |  |
| peak CK-MB, ng/ml |  |  |  |  |
|  | Total | 0.068 | 0.406 |  |
|  | Proximal LAD | 0.103 | 0.427 |  |
|  | Non-proximal LAD | 0.062 | 0.556 |  |
| peak TNI, ng/ml |  |  |  |  |
|  | Total | 0.139 | 0.086 |  |
|  | Proximal LAD | 0.199 | 0.120 |  |
|  | Non-proximal LAD | 0.110 | 0.298 |  |
| HsCRP, mg/L |  |  |  |  |
|  | Total | 0.209 | 0.010 |  |
|  | Proximal LAD | 0.313 | 0.013 |  |
|  | Non-proximal LAD | 0.087 | 0.413 |  |
| WBC, 10^9/^L |  |  |  |  |
|  | Total | 0.039 | 0.636 |  |
|  | Proximal LAD | 0.118 | 0.361 |  |
|  | Non-proximal LAD | -0.018 | 0.865 |  |
| HbA1c, % |  |  |  |  |
|  | Total | -0.056 | 0.491 |  |
|  | Proximal LAD | -0.014 | 0.915 |  |
|  | Non-proximal LAD | -0.112 | 0.292 |  |
| eGFR, ml/min/1.73m^2^ |  |  |  |  |
|  | Total | 0.026 | 0.749 |  |
|  | Proximal LAD | 0.022 | 0.866 |  |
|  | Non-proximal LAD | -0.015 | 0.890 |  |
| Creatinine, umol/L |  |  |  |  |
|  | Total | -0.004 | 0.962 |  |
|  | Proximal LAD | 0.012 | 0.928 |  |
|  | Non-proximal LAD | 0.046 | 0.664 |  |
| LDL-C, mmol/L |  |  |  |  |
|  | Total | -0.114 | 0.162 |  |
|  | Proximal LAD | -0.087 | 0.500 |  |
|  | Non-proximal LAD | -0.146 | 0.166 |  |
| ApoA1, g/L |  |  |  |  |
|  | Total | -0.117 | 0.152 |  |
|  | Proximal LAD | -0.077 | 0.551 |  |
|  | Non-proximal LAD | -0.122 | 0.249 |  |
| ApoB,g/L |  |  |  |  |
|  | Total | -0.122 | 0.133 |  |
|  | Proximal LAD | -0.128 | 0.321 |  |
|  | Non-proximal LAD | -0.174 | 0.164 |  |
| ApoE, g/L |  |  |  |  |
|  | Total | 0.078 | 0.336 |  |
|  | Proximal LAD | 0.091 | 0.483 |  |
|  | Non-proximal LAD | 0.088 | 0.409 |  |
| LP(a), mg/L |  |  |  |  |
|  | Total | 0.098 | 0.23 |  |
|  | Proximal LAD | 0.035 | 0.79 |  |
|  | Non-proximal LAD | 0.149 | 0.159 |  |
| LVEDV, ml |  |  |  |  |
|  | Total | -0.136 | 0.093 |  |
|  | Proximal LAD | -0.119 | 0.357 |  |
|  | Non-proximal LAD | -0.114 | 0.281 |  |
| LVESV, ml |  |  |  |  |
|  | Total | 0.006 | 0.938 |  |
|  | Proximal LAD | 0.08 | 0.539 |  |
|  | Non-proximal LAD | -0.049 | 0.644 |  |
| LVEDVi, ml/m^2^ |  |  |  |  |
|  | Total | -0.069 | 0.396 |  |
|  | Proximal LAD | -0.093 | 0.474 |  |
|  | Non-proximal LAD | -0.033 | 0.754 |  |
| LVESVi, ml/m^2^ |  |  |  |  |
|  | Total | 0.027 | 0.737 |  |
|  | Proximal LAD | 0.069 | 0.594 |  |
|  | Non-proximal LAD | 0.003 | 0.974 |  |
| LVEF, % |  |  |  |  |
|  | Total | -0.08 | 0.326 |  |
|  | Proximal LAD | -0.14 | 0.279 |  |
|  | Non-proximal LAD | -0.005 | 0.965 |  |

The p values were obtained from Pearson or Spearman test.

ApoA1, Apolipoprotein A1; ApoB, Apolipoprotein B; ApoE, Apolipoprotein E; CK-MB, creatine kinase-MB; eGFR, estimated glomerular filtration rate; HbA1c, glycated hemoglobin; HsCRP, [high sensitivity C-reactive protein](https://pubmed.ncbi.nlm.nih.gov/32679014/); LDL-C, low-density lipoprotein cholesterol; LP(a), Lipoprotein(a); LVEF, left ventricular ejection fraction; LVEDV, left ventricular end-diastolic volume; LVESV, left ventricular end-systolic volume; LVEDVi, left ventricular end-diastolic volume index; LVESVi, left ventricular end-systolic volume index; NT-proBNP, N-terminal pro-B type natriuretic peptide; TNI, troponin I; WBC, white blood cell.


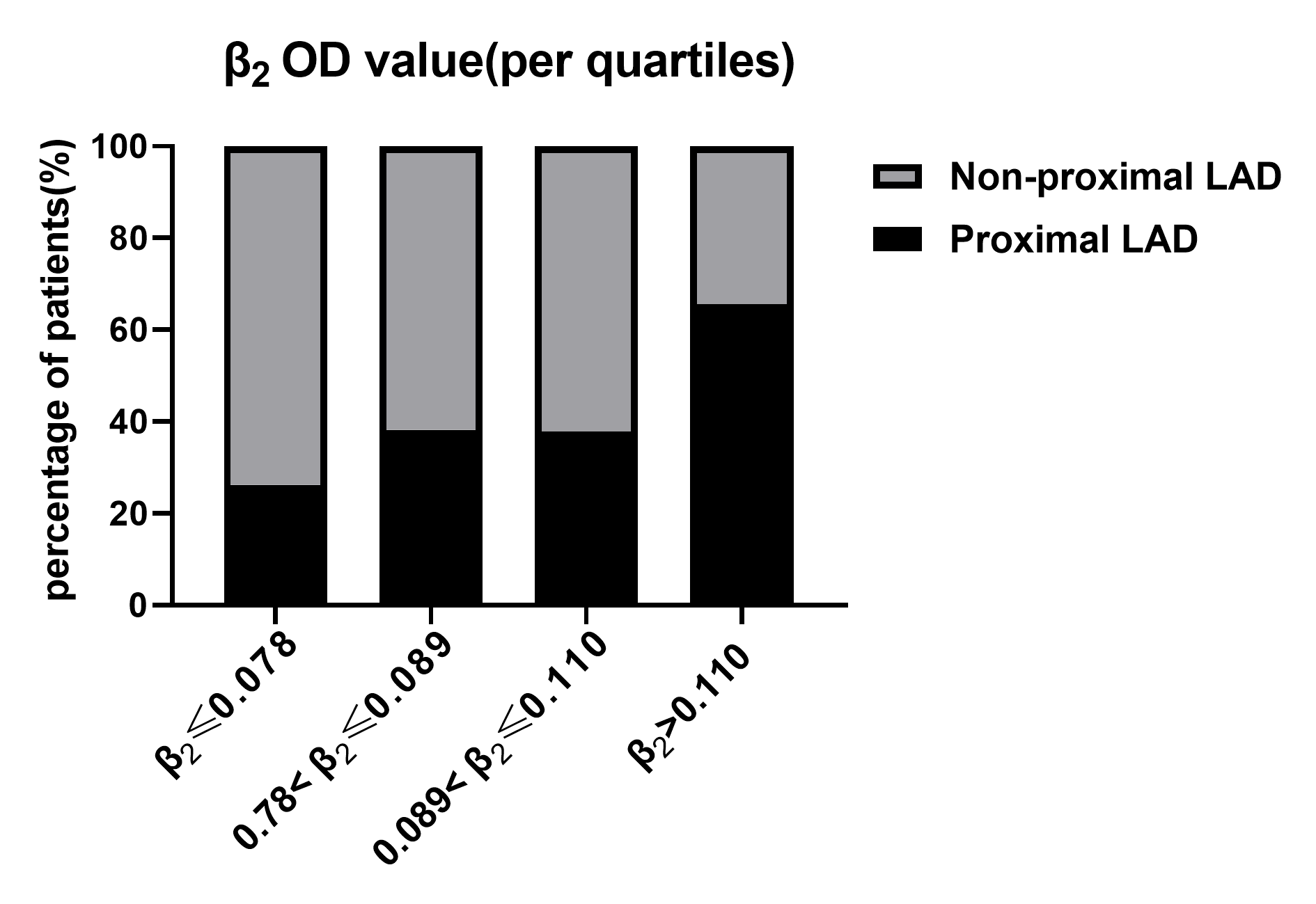


**Figure S1 Patients distribution according to β2-AA OD value**

Patients with proximal LAD are more frequently distributed in higher quartiles of β2-AA OD value. LAD, left anterior descending


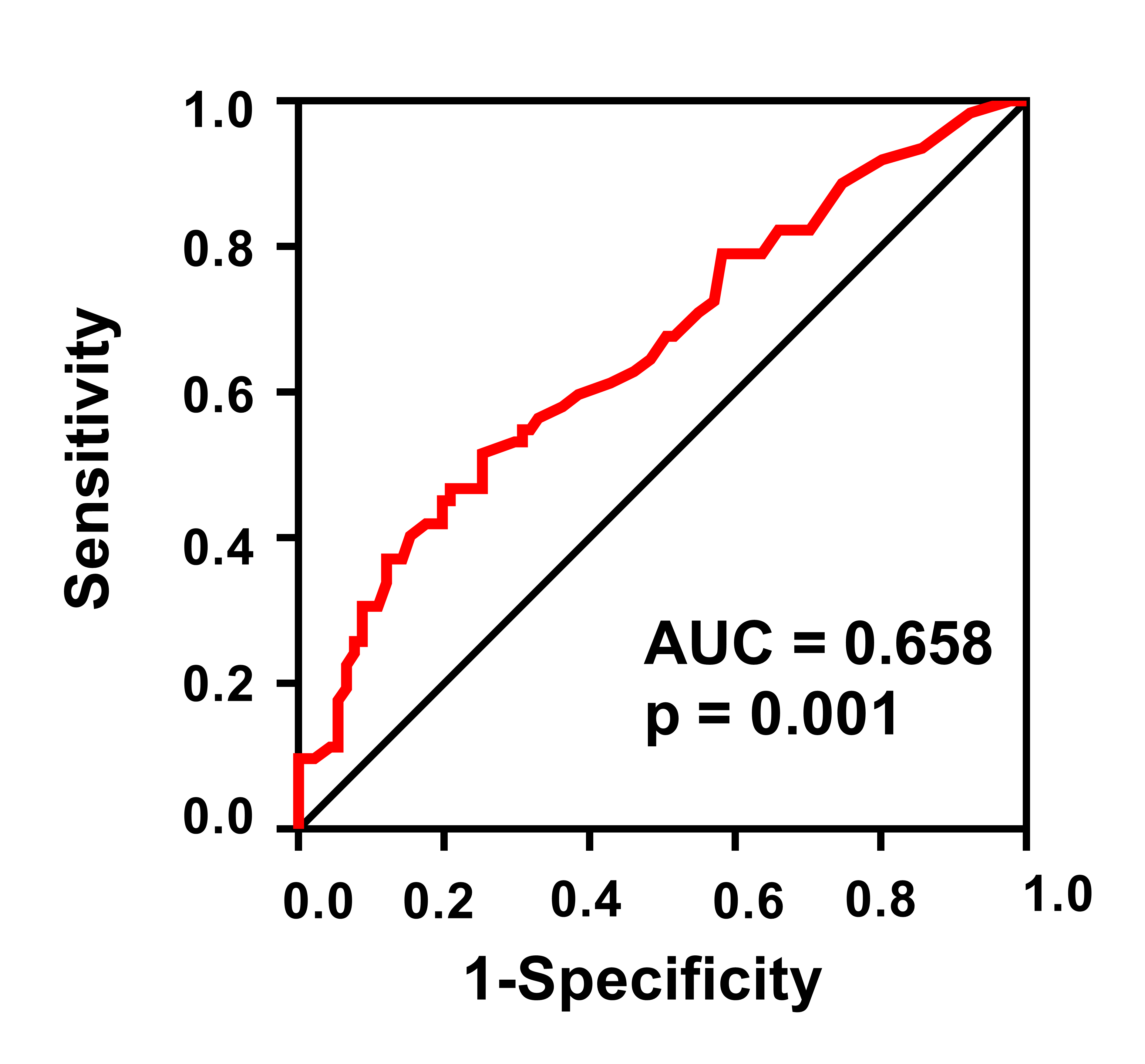


**Figure S2 The receiver operating characteristic (ROC) curve of the** **β2-AA OD value as a marker to predict proximal LAD lesion in STEMI patients**

The area under receiver operating characteristic (ROC) curve (AUC) of the β2-AA OD value for predicting the occurrence of proximal LAD lesion was 0.658 (95% CI 0.568-0.749; p = 0.001). LAD, left anterior descending
